# Trevor's Disease: A Literature Review regarding Classification, Treatment, and Prognosis apropos of a Case

**DOI:** 10.1155/2014/940360

**Published:** 2014-06-24

**Authors:** Georgios Arealis, Vassilios S. Nikolaou, Andrew Lacon, Neil Ashwood, Keith Hayward, Charalampos Karagkevrekis

**Affiliations:** ^1^Orthopedic Department, Queen's Hospital, Belvedere Road, Burton upon Trent, Staffordshire DE13 0RB, UK; ^2^2nd Department of Orthopaedics, School of Medicine, Athens University, Agia Olga Hospital, 3-5 Agias Olgas Street, 142 33 Athens, Greece

## Abstract

*Background*. Dysplasia epiphysealis hemimelica (DEH) is rare and its main characteristic is osteochondromas of the epiphysis of long bones.* Methods*. We report a case of DEH of the ankle in an 8-year-old boy that was resected in 2005. Additionally we collect all the reported cases of DEH. The literature is reviewed regarding the treatment, prognosis, long term function, and patterns and areas affected by DEH. *Results*. In our case no complications were noted and our patient remains asymptomatic. Reviewing the literature we found that 73 authors have reported 144 cases from 1926 to 2013. We propose and describe a new classification that correlates with prognosis. According to our classification DEH is classified as types 1 with single lower limb involvement, 2 with multiple lower limb, 3 with single upper limb, 4 with multiple upper limb, 5 with upper and lower limb, and 6 with spine. *Conclusions*. All single lesions should be followed up and if indicated a whole body nuclear bone scan can be useful in identifying the existence of multiple affected joints. Type 1 lesions have better prognosis than 2 and have less chances of developing OA even if not resected. Resection, even if partial, can be a successful treatment for DEH.

## 1. Background

Dysplasia epiphysealis hemimelica (DEH) was first described in 1926 by Mouchet and Belot as “tarsomegaly.” It is also known as “Trevor's disease” named after Trevor who reported in 1950 a series of 8 cases using the term “tarsoepiphyseal aclasis” [1]. It is rare condition and its main characteristic is osteochondromas of the epiphysis of long bones [2].

A case of DEH treated in our department allowed the literature to be reviewed regarding the treatment, prognosis, long term function, and patterns and areas affected by DEH. The diagnosis relies on the characteristic radiographic findings of DEH [3] that presents as an irregular lesion rising from the affected epiphysis [4, 5]. DEH has histological findings similar to benign osteochondroma [2, 6]. Asymptomatic lesions may be treated nonoperatively [7] but surgical intervention is very common [2].

According to Azouz et al., DEH is classified into three groups [8]. Following our review of the literature we believe that this classification is not adequate since it does not cover upper limb and simultaneous upper and lower limb involvement. Therefore based on our findings we propose and describe a new classification that correlates with return to normal activities.

## 2. Materials and Methods

### 2.1. Case Report

After obtaining written consent for publication we report the case of an 8-year-old boy who was referred to our clinic in 2004 because his parents observed that he was walking on the ball of his right foot and that his right calf was thinner than the other for 6 months. Other than this he was healthy. Both pregnancy and delivery were normal. The parents and the child denied any history of foot injury.

**Figure 1:**
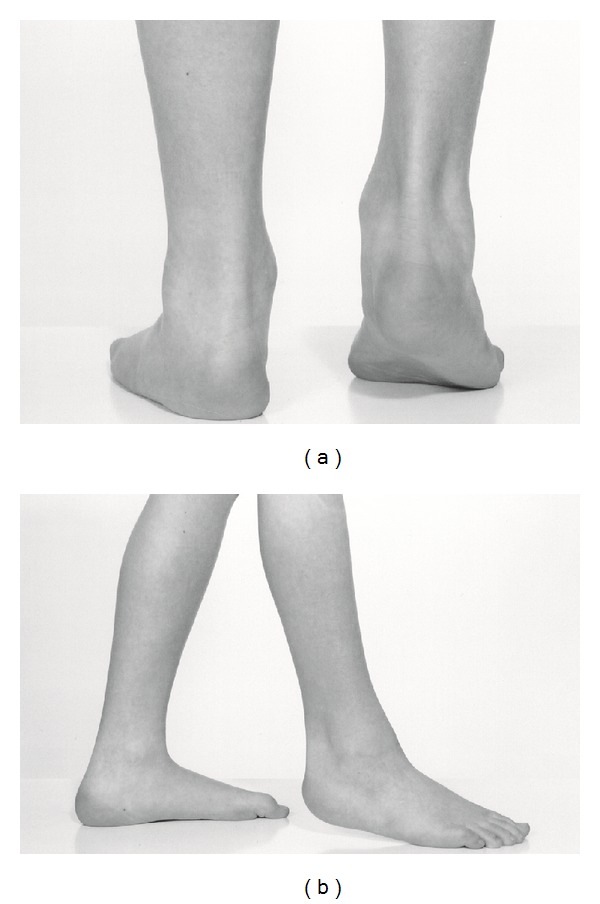
Right foot equinus and lack of dorsiflexion preoperatively: (a) posterior view, (b) lateral view.

On examination, he could not dorsiflex the right ankle and walked in equinus (Figure 1). His right calf was 3 cm thinner than the left. Plain radiographs of the right ankle showed a spur like lesion arising from the lateral distal tibial epiphysis and extending into the ankle joint that was blocking dorsiflexion. The fibula, the talus, and the foot were normal.

**Figure 2:**
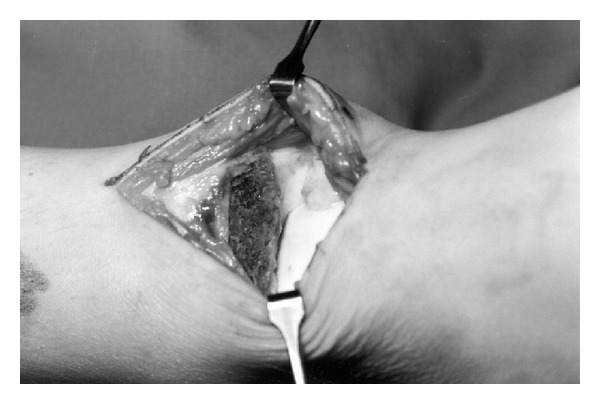
Intraoperative image of the removed lesion using a medial longitudinal incision.

**Figure 3:**
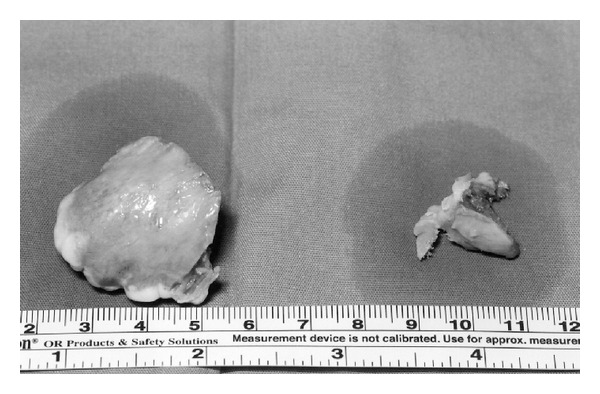
The two spurs that were removed from the anterior tibia. One larger medial (3.5 × 3.5 cm) and a smaller central (2 × 1 cm).

The clinical and radiologic findings were consistent with Trevor's disease (Figure 2). Since the spur blocked dorsiflexion, a decision for surgical exploration and removal was made. In 2005, under general anaesthesia and tourniquet the lesion was removed using a medial longitudinal incision (Figure 2). Using a broad osteotome two spurs were removed from the anterior tibia. A larger spur (3.5 × 3.5 cm) was removed from the anteromedial distal tibial epiphysis and a smaller one (2 × 1 cm) was removed from the anterior central (Figure 3). Intraoperative dorsiflexion and plantar flexion were checked and found to be almost normal.

The removed osteochondral fragments were sent for histological examination and the results showed that they were consistent with benign osteochondroma.

### 2.2. Literature Review

Using the US National Library of Medicine National Institutes of Health search engine (PubMed.gov) all cases with the keywords “dysplasia epiphysealis hemimelica,” “tarsomegaly,” “Trevor's disease,” “tarsoepiphyseal aclasis,” and “osteochondromas of the epiphysis” were collected using EndNote (version X6, Thomson Reuters). Data regarding the author name, year of publication, number of cases, side, and joints involved were recorded in a spreadsheet. When data regarding long term results were available, we recorded in a separate spreadsheet the range of movement, limb length difference, calf wasting, effect on daily activities, and main complain affecting function. All data were analysed using Excel (version 2010, Microsoft) and statistical analysis was performed using SPSS (version 20.0, IBM).

## 3. Results

### 3.1. Case Report

No intraoperative, immediate postoperative, or short term complications were noted. The child was followed up regularly for three years, until the age of 11 in our outpatient clinics. He was doing well with regard to his right ankle. At that time he had no pain and he had dorsiflexion of about 15 degrees from neutral and plantar flexion of 30 degrees from neutral position. The X-rays showed a small bony prominence on the anterior margin of the distal tibial epiphysis but he was not being affected by it. Therefore at that time he was discharged from the follow-up clinics with instructions to mobilize as able.

**Figure 4:**
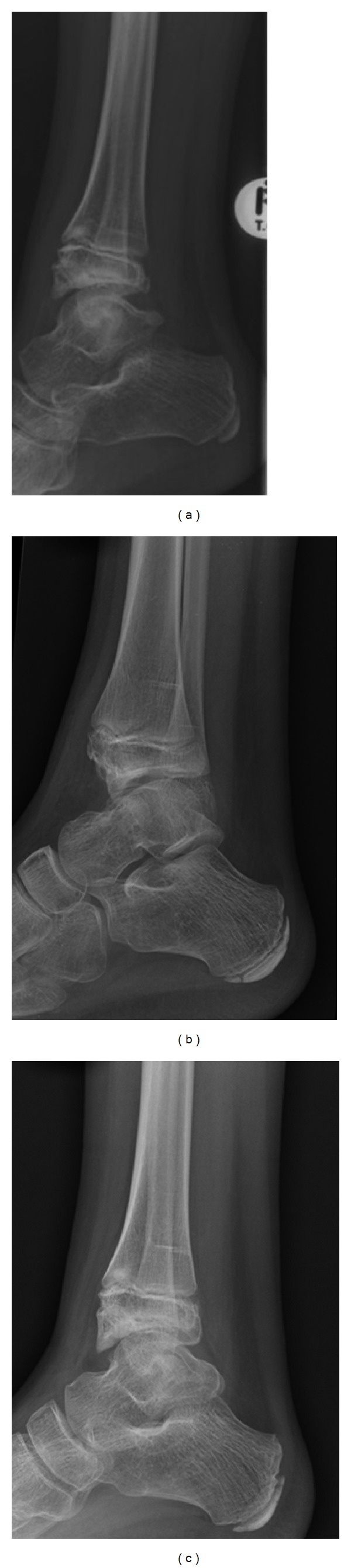
Serial X-rays: (a) preoperative in 2005, (b) postoperative in 2008, and (c) last followup in 2013, showing the lesion before and after its removal and the regrowth at the distal tibia.

At the age of 17, in 2013, he was seen again in our clinic because he noticed recently that he had some restriction of movement when going up stairs. On examination there was very little difference between his ankles' passive dorsiflexion although active dorsiflexion was decreased in the operated right ankle by 5 degrees. He had full plantar flexion in both ankles. An X-ray taken in 2013 showed recurrence of the lesion at the anterior distal lip of the tibia. However because of his excellent range of movement it was decided that surgery would not benefit him where he could see much improvement in his range of dorsiflexion (Figure 4).

### 3.2. Literature Review

Despite DEH's rarity, including our case, 73 authors have reported 144 cases from 1926 to 2013. There is a median of 2 cases reported every year since 1926. After reviewing the 144 published cases of DEH, excluding the 2 bilateral cases and including our case, adequate data for a meta-analysis was available for 138 (95.8%).

The lower limb is involved in 101 cases (73.2%), the upper in 33 (23.9%), both the upper and the lower in 3 (2.2%), and the spine in 1 (0.7%). Of the lower limb cases 50 (49.5%) involved a single joint and 51 (50.5%) multiple joints. Of the upper limb cases 30 (90.9%) involved a single joint and 3 (9.1%) multiple joints (Table 1).

The most commonly affected lower limb joint is the ankle (43.2%), followed by the knee (34.2%) and the hip (10.3%). The foot is affected in 11.5%. In single lower limb cases the ankle is involved in 44% (22 cases), the knee in 30% (15 cases), the hip in 14% (7 cases), and the foot in 12% (6 cases). In multiple lower limb cases the ankle is involved in 42.7% (41 cases), the knee in 36.5% (35 cases), the hip in 8.3% (8 cases), the sacroiliac joint in 1% (1 case), and the foot in 11.6% (11 cases) (Table 2).

The most commonly affected upper limb joint is the wrist (54.1%), followed by the elbow (13.5%) and the shoulder (13.5%). The hand is affected in 18.9%. In single upper limb cases the wrist is involved in 56.7% (17 cases), the elbow in 13.3% (4 cases), the shoulder in 10% (3 cases), and the hand in 20% (6 cases). In multiple upper limb cases the wrist is involved in 42.9% (3 cases), the elbow in 14.3% (1 case), the shoulder in 28.6% (2 cases), the sacroiliac joint in 1% (1 case), and the hand in 14.3% (1 case) (Table 3).

Simultaneous upper and lower limb involvement on the same side is very rare (3 cases, 2.2%). Similarly there has been only one report of DEH of the spine (0.7%).

The surrounding soft tissues can be in danger, especially the nerves, and there is one report of ulnar nerve compromise [9]. All the epiphysis of the affected joint can be involved, including the sacroiliac joint [10], the acetabulum [11], and the patella [4].

Following analysis of joint and limb involved we propose a classification based on the number of joints involved and whether the upper or lower limb is affected. According to our classification DEH is classified as types 1 with single lower limb involvement, 2 with multiple lower limb involvement, 3 with single upper limb, 4 with multiple upper limb, 5 with upper and lower limb involvement, and 6 with spine (Table 4).

Types 1 to 3 are the most common and are 94.5% of all cases. More specifically type 1 (lower single) is 36.2% of all cases, type 2 (lower multiple) is 37%, and type 3 (single upper) is 21.7%. Types 4 to 6 are rare. Type 4 (multiple upper) is 2.2%, type 5 (upper and lower) is 2.2%, and type 6 (spine) is 0.7%.

From the 138 reported cases we managed to collect long term results for 26 cases, including ours [6, 11, 12]. The median follow-up time was 8.5 (range: 1–37 years). The treatment was resection in 23 (88.5%). Of them 16 were type 1 (61.5%), 9 type 2 (34.5%), and 1 type 3 (4%). Almost all patients (24, 92.3%) had equal limb length, 19 (73.1%) had muscle wasting, and 15 (57.7%) had full range of movement. No malignancy was reported. Full daily activities were possible for 20 (76.9%) of the patients but 15.4% (4 cases) resulted in knee or ankle osteoarthritis and 7.7% (2 cases) needed arthrodesis of the ankle joint. Using SPSS (IBM statistics, version 20) Pearson's correlation was used to identify relations between the various variables. Significant correlation between the type of DEH and daily activities (*P* = 0.045) was found. Following this we repeated the analysis using chi2 and focusing only on type 1 and type 2 cases. We did not find significant correlation between resection and osteoarthritis (*P* = 0.383) or the need for arthrodesis (*P* = 0.085). No significant correlation between type and osteoarthritis (OA) was shown (*P* = 0.076). We must note, however, that only 1 out of 16 (6.2%) patients with type 1 DEH resulted in OA in contrast to 3 out of the 9 patients with type 2 (33.3%) and the results may be influenced by the small number of cases. Again a very strong correlation between type and return to daily activities (*P* = 0.006) was evident. Of the 16 type 1 patients, 15 (93.7%) had full activities in contrast to only 4 out of 9 (44.4%) of type 2 (Table 5). No other significant correlations were evident.

## 4. Discussion

### 4.1. Etiology

DEH results from an abnormal control of cell proliferation at the effected epiphysis. The actual cause of the abnormality has not been identified, but when this occurs the overgrowth follows enchondral ossification resulting in bone overgrowth with a cartilage cap that projects into the adjacent joint [2, 12].

DEH is sporadic and neither genetic component in the aetiology nor any common environmental factor has been found in the reported series [2, 12]. This was similar in our case and no genetic or environmental predisposition was identified. Hensinger et al. reported 7 cases of familial dysplasia epiphysealis with epiphyseal chondromas and osteochondromas in 12 generations of one family [14]. Since then, three more cases of familial dysplasia epiphysealis have been reported [15, 16]. All of these cases are dominant carpotarsal osteochondromatosis (DCO). DCO has an autosomal dominant inheritance and bilateral involvement of joints and is a different entity from DEH even though both have similar epiphyseal chondromas and osteochondromas [15].

### 4.2. Incidence

Dysplasia epiphysealis hemimelica belongs to the group of skeletal osteochondromas (OC) or osteocartilaginous exostoses. Even though OC is the most common of all benign bone tumours and represents 10% to 15% of all bone tumours, DEH is rarer. DEH has a reported incidence of 1 : 1.000.000 [17, 18]. Despite DEH's rarity, including our case, 73 authors have reported 144 cases from 1926 to 2013. There is a median of 2 cases reported every year since 1926. Apart from Trevor's original paper three more significant review papers have been published. Rosero et al. reviewed most lower limb cases up to 2007 [5], Vogel et al. collected all upper limb cases up to 2009 [19], and Fairbank collected all the known cases up to 1956 [13].

The condition is 3 times more frequent in males than in females [9]. Unilateral involvement is very common and hence the term hemimelica. Of the 144 reported cases only 2 (1.3%) were bilateral [20]. The lesions affect the medial epiphysis twice as often [21]. Even though our case involves the lateral epiphysis, that is more uncommon, the rest of its presentation is typical; it involved a young boy and a single ankle joint. The age of 8 is also typical for first presentation. Most of the cases appear between the ages of 2 and 8 but cases have been reported in patients from 2 months to 40 years old [8, 18, 22–24].

### 4.3. Symptoms and Diagnosis

The clinical manifestations of DEH, irrespective of the involved joints, consist of functional impairment and limitation of range of movement, deformity and swelling, and in some cases pain and wasting of the muscles that move the affected joint. Gait abnormalities or limb length discrepancy can also be present [19, 25, 26]. Simple radiographs are very important for the diagnosis of DEH [3], and the radiographic findings are characteristic. In early stages it presents as an irregular lesion rising from the affected epiphysis, and then gradually calcification centres appear and grow. Eventually it ossifies and usually becomes confluent with the underlying bone [4, 5]. Sometimes it can be difficult to differentiate between DEH and parosteal osteosarcoma and osteoblastoma, especially in the early stages [6, 27] and if the talus is affected, since it may remain separated from the host bone [28]. CT is very useful in identifying calcification or ossification within the DEH lesion and to define cortical and medullar continuity between the lesion and the adjacent bone [27, 29]. Also, 3-dimensional reconstructions of the CT images can be helpful in the preoperative planning [23].

MRI is useful in determining the size of the cartilaginous part of the DEH lesion. Additionally it provides information regarding the involvement of the epiphysis, the surrounding soft tissues, and the joint [4, 29]. The DEH mass has a low to intermediate signal on T1 weighted images and high signal on T2. [6] Once the lesion matures and fully ossifies, the signal has been reported to be low on both T1 and T2 weighted images [30]. The affected joint usually is irregular and oedema of both the bone marrow and the surrounding soft tissue may be evident [6].

The DEH lesions have an increased uptake in nuclear bone scans. A whole body skeletal scintigraphy can be used if one lesion is found in order to determine the number of joints and limbs involved and thus to define the DEH type [31].

### 4.4. Pathology Findings

DEH lesions have histological findings similar to benign osteochondroma; alike were the findings of our case. There is a cap of disorganized hyaline cartilage over a mass of tissue with enchondral ossification of varying degrees and progression to cancellous bone [2, 6].

### 4.5. Classification

According to Azouz et al. DEH is classified into 3 groups: localized that affects only one epiphysis, classic that affects more than one epiphysis in the same limb, and generalized that involves the entire lower limb [8]. Following our review of the literature we believe that this classification does not fully describe upper limb and simultaneous upper and lower limb involvement. Additionally, a classification scheme that is based on the joints involved would be simpler and more useful in everyday clinical practice, especially if it could be used to predict the outcome of DEH. Therefore we propose that DEH should be classified as types 1 with single lower limb involvement, 2 with multiple lower limb involvement, 3 with single upper limb, 4 with multiple upper limb, 5 with upper and lower limb involvement, and 6 with spine (Table 4).

### 4.6. Treatment and Prognosis

Even though it is reported that asymptomatic lesions may be treated nonoperatively [7], surgical intervention is very common, especially compared to solitary osteochondromas, because often the adjacent joint is involved [2]. If the lesion is treated nonoperatively, careful followup is indicated in order to evaluate the progression of the lesion. Massive ossification of the hypertrophic cartilaginous areas [26] within 4 years and early osteoarthritis of the ankle [18] in 2 years have been reported.

Surgical treatment consists of removal of the lesion from the affected epiphysis. Incompletely removed lesions usually dissolve and cause no problems [6, 13] but reports of local recurrence also exist [7]. Therefore an attempt to fully remove the lesion without injuring the epiphysis should be made. Corrective osteotomies may be necessary to treat coexisting deformities [12].

Surgical excision warrants very good long term results [6, 11, 12]. Almost all patients have equal limb length and 2/3 have full range of movement. Muscle wasting, though, persists and is found in 70% of the patients in the long term. No malignancy was reported. In the long term, full daily activities were possible in most of the patients but 15% may result in knee or ankle osteoarthritis and 7.7% will need arthrodesis of the ankle joint. The incidence of osteoarthritis increases to 17.6% in the 17 cases who had over 6 years of followup (median: 13 and range: 6–34 years). Overall type 1 has better results and compared to type 2 return to daily activities is significantly better (*P* = 0.045). Additionally type 1 patients are less likely to develop osteoarthritis of the affected joint.

## 5. Conclusions

In DEH patients if a lower limb lesion is found, then there is almost a 50% chance that this is type 1 (single lower limb). Also, if an upper limb lesion is found, there is an 85.7% chance that this is type 3 (single upper limb). This means that single joint involvement is more common in upper limb than in lower limb. All single lesions should be followed up and if any clinical suspension exists a whole body nuclear bone scan can be useful in identifying the existence of multiple affected joints.

Type 1 lesions have better prognosis than 2 and have less chances of developing OA even if not resected. Resection, even if partial, can be a successful treatment for DEH.

## Figures and Tables

**Table 1:** Percentage and number of joints affected by DEH in the cases reported in the literature.

Limb involved	Cases	%	%	Cases	Joints involved
Lower limb	101	73.2	49.5	50	Single lower
			50.5	51	Multiple lower
Upper limb	33	23.9	90.9	30	Single upper
			9.1	3	Multiple upper
Upper and lower	3	2.2			
Spine	1	0.7			

Total	138	100			

**Table 2:** Lower limb joints affected by DEH reported in the literature.

	Cases	%	Sacroiliac	Hip	Knee	Ankle	Foot	
Lower single	50	36.2	0	7	15	22	6	Cases
			0.00	14.00	30.00	44.00	12.00	%
Lower multiple	51	37.0	1	8	35	41	11	Cases
			1.04	8.33	36.46	42.71	11.46	%

Lower total	101	73.2	1	15	50	63	17	Cases
			0.68	10.27	34.25	43.15	11.64	%

**Table 3:** Upper limb joints affected by DEH reported in the literature.

	Cases	%	Shoulder	Elbow	Wrist	Hand	
Upper single	30	21.7	3	4	17	6	Cases
			10.00	13.33	56.67	20.00	%
Upper multiple	3	2.2	2	1	3	1	Cases
			28.57	14.29	42.86	14.29	%

Upper total	33	23.9	5	5	20	7	Cases
			13.51	13.51	54.05	18.92	%

**Table 4:** New classification, number, and percentage of joints affected: lower or upper limb, single or multiple.

	Cases	%	Type	
Lower single	50	36.23	1	Common
Lower multiple	51	36.96	2	Common
Upper single	30	21.74	3	Common
Upper multiple	3	2.17	4	Rare
Upper and lower	3	2.17	5	Rare
Spine	1	0.72	6	Rare

	138	100		

**Table 5:** Long term results of the reported DEH cases.

Author	Years of followup	ROM	Limb length	Calf wasting	Daily activity	Site	Type	Sex	Age at diagnosis in years	Years from diagnosis to surgery	Resected	Arthro-desis	OA
Bahk et al. [6]	11	Full	Normal	Yes	Normal	Knee	1	M	6	0.5	Yes	No	No
Bahk et al. [6]	8	Full	Normal	Yes	Normal	Wrist	3	F	11	6	Yes	No	No
Bahk et al. [6]	9	Full	Normal	Yes	Normal	Ankle	1	F	4	0.5	Yes	No	No
Bahk et al. [6]	6	Full	Normal	Yes	Normal	Knee, ankle	2	M	6	0.5	Yes	No	No
Bahk et al. [6]	3	Full	Normal	Yes	Normal	Ankle	1	F	2	0.5	Yes	No	No
Bahk et al. [6]	4	Full	Normal	Yes	Normal	Knee	1	M	3	2	Yes	No	No
Bahk et al. [6]	1	Full	Normal	Yes	Normal	Knee, ankle, foot	2	M	8	4	Yes	No	No
Bahk et al. [6]	2	Full	Normal	Yes	Normal	Ankle	1	M	7	0.5	Yes	No	No
Connor et al. [12]	21	Restricted	Normal	Yes	Normal	Ankle	1	M	8	1	Yes	No	No
Connor et al. [12]	18	Full	Normal	No	Normal	Hip, knee, ankle	2	M	2	0	No	No	No
Connor et al. [12]	13	Restricted	Normal	Yes	Normal	Ankle	1	M	3	3	Yes	No	No
Connor et al. [12]	10	Full	Normal	No	Normal	Knee	1	M	5	1	Yes	No	No
Connor et al. [12]	9	Restricted	Normal	Yes	Normal	Ankle	1	M	0.8	6.2	Yes	No	No
Connor et al. [12]	6	Restricted	Reduced	Yes	Reduced	Knee, ankle, foot	2	F	1	4	Yes	No	No
Connor et al. [12]	4	Restricted	Reduced	Yes	Normal	Ankle	1	F	1.5	0	No	No	No
Connor et al. [12]	3	Full	Normal	Yes	Normal	Ankle	1	M	8	2	Yes	No	No
Connor et al. [12]	2	Restricted	Normal	Yes	Reduced	Knee, ankle	2	M	1.5	1.5	Yes	No	No
Fairbank [13]	28	Full	Normal	No	Normal	Knee	1	M	3	1	Yes	No	No
Fairbank [13]	26	Full	Normal	No	Normal	Knee, ankle	2	M	14	9	Yes	No	No
Fairbank [13]	16	Restricted	Normal	Yes	Reduced	Ankle	1	F	6.5	0	No	Yes	Yes
Metcalfe [10]	2	Restricted	Normal	No	Reduced	Ankle, foot	2	M	0.9	8.1	Yes	Yes	Yes
Trevor [1]	37	Full	Normal	No	Normal	Knee	1	M	8	0	Yes	No	No
Trevor [1]	34	Restricted	Normal	Yes	Reduced	Knee, ankle, foot	2	F	0.9	1.1	Yes	No	Yes
Trevor [1]	23	Restricted	Normal	No	Reduced	Knee, ankle	2	M	4.5	0.5	Yes	No	Yes
Tschauner et al. [11]	4	Full	Normal	Yes	Normal	Hip	1	M	7	0.5	Yes	No	No
Ours	8	Restricted	Normal	Yes	Normal	Ankle	1	M	8	1	Yes	No	No
